# Anti-Inflammatory Potential of Seasonal Sonoran Propolis Extracts and Some of Their Main Constituents

**DOI:** 10.3390/molecules28114496

**Published:** 2023-06-01

**Authors:** Mayra A. Mendez-Encinas, Dora Valencia, Jesús Ortega-García, Elizabeth Carvajal-Millan, José C. Díaz-Ríos, Pablo Mendez-Pfeiffer, Cinthia M. Soto-Bracamontes, Adriana Garibay-Escobar, Efrain Alday, Carlos Velazquez

**Affiliations:** 1 Department of Chemical Biological and Agropecuary Sciences, University of Sonora, Avenida Universidad e Irigoyen, Caborca 83621, Mexico; 2 Research Center for Food and Development, CIAD, A.C. Carretera Gustavo Enrique Astiazaran Rosas No. 46, Hermosillo 83304, Mexico; 3 Department of Chemistry-Biology, University of Sonora, Blvd. Luis Encinas y Rosales S/N, Hermosillo 83000, Mexicoefrain.alday@unison.mx (E.A.);

**Keywords:** Sonoran Desert propolis, in vitro anti-inflammatory activity, seasonality, nitric oxide

## Abstract

Biological properties of Sonoran propolis (SP) are influenced by harvest time. Caborca propolis showed cellular protective capacity against reactive oxygen species, which might be implicated in anti-inflammatory effects. However, the anti-inflammatory activity of SP has not been investigated so far. This study investigated the anti-inflammatory activity of previously characterized seasonal SP extracts (SPE) and some of their main constituents (SPC). The anti-inflammatory activity of SPE and SPC was evaluated by measuring nitric oxide (NO) production, protein denaturation inhibition, heat-induced hemolysis inhibition, and hypotonicity-induced hemolysis inhibition. SPE from spring, autumn, and winter showed a higher cytotoxic effect on RAW 264.7 cells (IC_50_: 26.6 to 30.2 µg/mL) compared with summer extract (IC_50_: 49.4 µg/mL). SPE from spring reduced the NO secretion to basal levels at the lowest concentration tested (5 µg/mL). SPE inhibited the protein denaturation by 79% to 100%, and autumn showed the highest inhibitory activity. SPE stabilized erythrocyte membrane against heat-induced and hypotonicity-induced hemolysis in a concentration-dependent manner. Results indicate that the flavonoids chrysin, galangin, and pinocembrin could contribute to the anti-inflammatory activity of SPE and that the harvest time influences such a property. This study presents evidence of SPE pharmacological potential and some of their constituents.

## 1. Introduction

Inflammation is a protective mechanism of the organism in response to external and internal stimuli caused by mechanical damage or infections by pathogens. During the early stages of the inflammation process, macrophages activate the release of pro-inflammatory cytokines, including tumor necrosis factor alpha (TNF-α), interleukin 1β (IL-1 β), and interleukin 6 (IL-6) [1]. Macrophages are also responsible for stimulating the activation of nuclear factor kappa B (NF-kB), which has a crucial role as a mediator in the expression of genes involved in the apoptosis process as well as promoting inflammatory and pro-inflammatory cytokine expressions [2]. Another inflammatory mediator is nitric oxide, which, when produced in large amounts by inflamed endothelial cells, can cause tissue damage [3]. In certain circumstances, if inflammation is not controlled and chronic inflammation is produced, it can have consequences for the body and lead to the development of various diseases, such as diabetes, rheumatoid arthritis, cardiovascular and respiratory diseases, autoimmune diseases, and cancer. The conventional treatment to control inflammation involves administrating steroidal and non-steroidal anti-inflammatory drugs [4]. However, these drugs are not always effective in treating all types of inflammation and may result in undesirable side effects causing the patient not to continue the treatment. This situation has led to an increasing search for novel alternatives for treating inflammation. Mainly, interest has focused on identifying compounds with anti-inflammatory activity extracted from natural sources, as they can offer certain advantages compared with synthetic drugs, such as the low incidence of adverse effects in the patient. In this sense, propolis is considered one of the most promising natural substances due to its significant anti-inflammatory potential.

Propolis is a resinous substance produced by honeybees (*Apis mellifera*) from various botanical sources [5]. This material has been widely used in traditional medicine due to its nutritional and therapeutic properties. Antioxidant, antimicrobial, antiviral, immunomodulatory, antiproliferative, and anti-inflammatory activity are among their most documented properties [6,7,8,9,10]. Propolis represents an important source of bioactive compounds, among which flavonoids, terpenes, and derivatives of phenolic acids stand out. The biological properties of propolis are directly related to its chemical composition, which, in turn, is influenced by various factors, such as the bee species, the type of vegetation, the geographical area, and the harvest season [11,12,13].

Previous studies have shown a close relationship between seasonality and the chemical composition of propolis, which, in turn, has a meaningful impact on its biological properties. Sonoran propolis (SP) has demonstrated an important antiproliferative effect against various cancer cell lines, antimicrobial activity against certain strains of clinical interest, and a great capacity to inhibit free radicals [14,15,16]. Likewise, it has been possible to identify those constituents that could be mainly responsible for the pharmacological potential of propolis. Caffeic acid phenethyl ester (CAPE), galangin, xanthomicrol, and chrysin showed significant antiproliferative activity against various cancer cell lines [16]. CAPE, one of the compounds identified in SP, showed a remarkable protective effect against oxidative stress in two murine cell lines (B-lymphoma and macrophages) [17]. Recently, the ability of SP (Caborca) to inhibit the production of intracellular reactive oxygen species (ROS) induced by H_2_O_2_ was demonstrated, which resulted in its being modulated by the collection time of the samples [13]. Moreover, SP (Ures) has been demonstrated to exert a modulatory effect by inhibiting the pro-inflammatory cytokine TNF-α and stimulating the anti-inflammatory cytokine IL-10 in human monocytes [6]. However, the anti-inflammatory activity of SP has not been evaluated so far. This work aimed to evaluate the in vitro anti-inflammatory activity of SP extracts (SPE) harvested in different seasons of the year and some of their main constituents (SPC).

## 2. Results and Discussion

### 2.1. Antiproliferative and Cytotoxic Activities of Seasonal SPE and SPC on RAW 264.7 Cells

The MTT assay was used to determine the capacity of seasonal SPE and SPC to inhibit the proliferation of RAW 264.7 cells and to identify the concentrations at which the extracts could test for an anti-inflammatory effect. Table 1 shows the IC_50_ values of SPE and SPC in RAW 264.7 cells. The SPE collected in each of the seasons of the year (spring, S; summer, M; autumn, A; and winter, W) induced an antiproliferative effect at low extract concentrations in the RAW 264.7 cell line. The SPE from S (26.6 ± 2 µg/mL), A (26.5 ± 1.5 µg/mL), and W (30.2 ± 2 µg/mL) showed a more significant inhibitory effect on cell proliferation compared with the samples from M (49.4 ± 1.8 µg/mL). All the extracts were able to inhibit cell proliferation by 50% at concentrations below 30 µg/mL, meeting the criteria established by the National Cancer Institute of the United States for compounds with antiproliferative activity [18]. However, no significant difference (*p* ≤ 0.05) was found among the IC_50_ values of the S, A, and W samples. Several reports have documented that harvest season significantly influences the antiproliferative activity of propolis [8,12,13]. A previous study evaluated the antiproliferative effect of propolis extracts from Caborca, Sonora, harvested during the four seasons of the year on the M12.C3.F6 cell line and found that the sample harvested during autumn exhibited the highest antiproliferative effect, with an IC_50_ value of 5.9 µg/mL [13]. Likewise, the propolis collected during the spring season in the region of Ures, Sonora, showed higher antiproliferative activity in the M12.C3.F6 cell line in comparison with the other seasons [8]. These studies coincide with the results obtained in the present study, where the SPE from S and A showed more significant antiproliferative activity. Climatic variations, such as rain, drought, and high temperatures, influence the chemical composition of propolis by modulating the biosynthesis of certain metabolites, which, in turn, impacts its biological properties [11]. In the Brazilian red propolis, it was found that the rainy season favored the biosynthesis of flavonoids, while during the dry season, the concentrations of guttiferone monoterpenoids increased [11].

Several studies have shown that the biological activities of propolis are dependent on its chemical composition. Hernandez et al. [16] indicated that some compounds present in propolis from some regions of Sonora (Ures, Álamos, and Caborca) could be partly responsible for its antiproliferative activity. The compounds CAPE, xanthomicrol, galangin, and chrysin, present in SP, had significant antiproliferative activity in different cell lines, with the RAW 264.7 murine cell line being the most sensitive to the extracts. The polyphenolic composition of the methanolic SPE collected over all the seasons of the year was previously characterized by HPLC-UV-DAD and recently reported, showing that pinocembrin was the most abundant component in the seasonal samples (12.8–45.8 µg/mg), followed by pinobanksin-3-O-acetate (5–24.9 µg/mg), galangin, and chrysin. SPE from S and A presented similar contents of the compounds chrysin (˂5 µg/mg) and galangin (˂5 µg/mg), while the SPE from W showed a similar content for chrysin and a slightly higher one for galangin (7.4 µg/mg) [13]. The antiproliferative activity of some of the polyphenolic constituents (chrysin, galangin, and pinocembrin) identified in SPE was also assessed to determine its contribution to the antiproliferative activity of the extracts (Table 1). The three components evaluated showed similar antiproliferative activity in RAW 264.7 cells, with IC_50_ values of 56.2, 52.4, and 56.16 µM for chrysin, galangin, and pinocembrin, respectively. According to this information, the antiproliferative activity of SPE could be due to a synergistic mechanism of action of several of its constituents, and even other unidentified components could be contributing to its antiproliferative activity.

**Figure 1:**
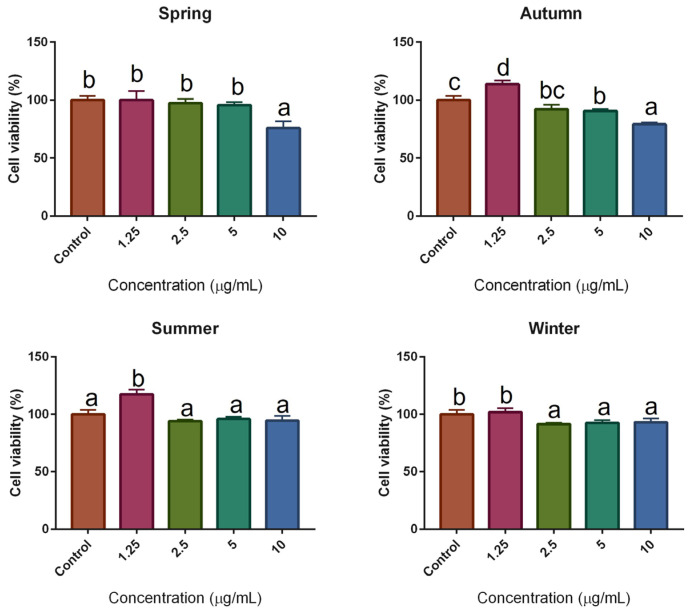
Cytotoxic effect of seasonal SPE on RAW 264.7 cells. Bars with different superscripts indicate statistical differences (*p* ≤ 0.05). All values represent the mean ± SD of at least three independent experiments performed in triplicate (*n* = 3).

Seasonal SPE showed that they can induce damage to RAW 264.7 cells after 24 h of incubation. During the antiproliferative assays, large cells were observed without a defined morphology, agglutination, or cell lysis. The four samples managed to induce cell damage and lysis, which was dependent on the concentration since the lower the concentration, the lower the number of damaged cells. These observations coincide with the results obtained in the antiproliferative assays and suggest a possible mechanism of inhibitory action of cell proliferation. The seasonal SPE did not show a cytotoxic effect at concentrations lower than 10 µg/mL (cell viability ≥ 80%) (Figure 1), while the SPC did not affect the viability of RAW 264.7 cells at concentrations lower than 10 µM. These concentrations were selected to carry out the anti-inflammatory activity assays to avoid cytotoxic concentrations and to guarantee that the effects observed in the experiments are attributed only to an inflammatory response and not to cell death.

### 2.2. Effect of SPE and SPC on NO Production

**Figure 2:**
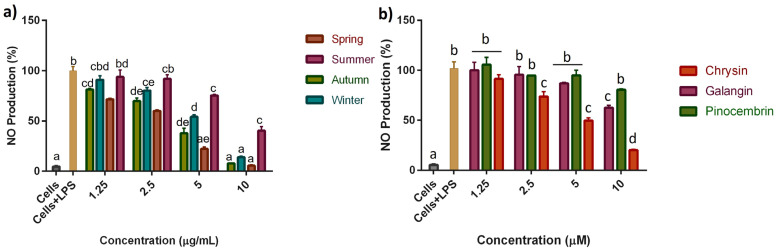
Effect of (**a**) seasonal SPE and (**b**) SPC on NO production of RAW 264.7 cells activated with LPS (1 µg/mL). Values are expressed as means ± SD of three independent experiments performed in triplicate (*n* = 3). LPS: lipopolysaccharide. Data were analyzed by one-way ANOVA, followed by Tukey’s multiple comparison test. Bars with different letters indicate statistical differences (*p* ≤ 0.05) compared with control groups (Cells and Cells + LPS).

The potential anti-inflammatory effect of seasonal SPE and SPC was evaluated using the Griess reagent by measuring their capability to reduce the NO levels produced by LPS-stimulated RAW 264.7 cells. All treatments reduced the NO production in RAW 264.7 cells stimulated with LPS for 24 h, showing a dose-dependent effect (Figure 2). Notably, seasonality was shown to have an impact on the anti-inflammatory activity of SPE. The SPE from A and W managed to decrease the NO levels to basal levels at a concentration of 10 µg/mL, while the SPE from S showed a greater anti-inflammatory effect by decreasing the NO production to basal levels at a concentration of 5 µg/mL. According to the calculated IC_50_ values, the most effective extract in reducing the NO production was S, with the lower IC_50_ value (3.35 ± 0.3 µg/mL), followed by A, W, and finally M, which exhibited the higher IC_50_ value (8.68 ± 0.01 µg/mL) (Table 2). Previous studies showed that Brazilian red propolis at a concentration of 50 µg/mL decreased NO production by 78% in LPS-activated RAW 264.7 macrophages [19]. In this work, the SPE from S, A, and W decreased NO production levels between 86% and 95% at a concentration of 10 µg/mL. The results suggest that seasonal SPE could have an anti-inflammatory effect, partly related to the inhibition of NO production by macrophages. Likewise, the harvest season’s influence on the anti-inflammatory properties of propolis has been established.

NO is a signaling molecule that plays an essential role during the inflammation process. The excessive production and release of this molecule are widely associated with various diseases. NO is generated in biological tissues by the enzyme-inducible NO synthase (iNOs), which metabolizes arginine to citrulline, with the subsequent formation of NO [20]. In this way, the search for molecules that could act as scavengers of NO or inhibitors of its production are of interest for the development of agents with anti-inflammatory action, as they could be used to mitigate the propagation of inflammation by NO [21]. A recent study found that propolis decreases the levels of certain molecules, such as hydroxyarginine, an intermediate in the production of NO from arginine, and citrulline, which is a product that remains after the formation of NO [22].

The anti-inflammatory mechanism of propolis has been linked to its complex chemical composition. Flavonoids can inhibit the enzyme-inducible cyclooxygenase synthase and iNOs by binding to the PPAR-γ receptor on macrophages [23], so it is likely that their mechanism of action is partly attributed to its favoring the activation of this receptor [22]. To determine the contribution of flavonoids in the anti-inflammatory activity of the seasonal SPE, the anti-inflammatory activity of the compounds chrysin, galangin, and pinocembrin (some of the flavonoids identified in these extracts) was evaluated (Figure 2 and Table 2). The best inhibitory activity for NO production was obtained for the flavone chrysin, as evidenced by the low IC_50_ value (5.8 ± 0.2 µM) in comparison with the flavanol galangin and the flavanone pinocembrin, both showing IC_50_ values higher than 10 µM. Previous studies have documented that flavones and flavonols exhibit a greater capability to inhibit NO production than flavanones [24,25]. According to these results, mainly the flavonoids chrysin and galangin could be partly contributing to the anti-inflammatory activity of the seasonal SPE, even when they are present in the extracts in a lower concentration (˂7.4 µg/mg) than that of pinocembrin (>12.8 µg/mg) [13]. The flavonoids chrysin, pinocembrin, galangin, and pinobanksin reduced the NO secretion in LPS-induced H9c2 cells, and in particular, chrysin was able to reduce the NO levels by 63% at 25 µM [26]. However, the contribution of other unidentified compounds in the extracts to their anti-inflammatory activity should be considered.

### 2.3. Effect of SPE and SPC on Protein Denaturation

Protein denaturation refers to a process in which the proteins lose their quaternary, tertiary, and secondary structures due to exposure to external stress or compounds, which generally leads to a loss of their biological functions [27]. Denaturation of tissue proteins is one of the well-documented causes of inflammatory and arthritic diseases. Production of autoantigens in certain arthritic diseases may be due to the denaturation of proteins in vivo [28,29]. For this reason, tissue protein denaturation may be considered as a marker for inflammatory and arthritic diseases [27]. Agents that can prevent protein denaturation could be of great interest in developing anti-inflammatory drugs. It has been reported that one of the features of several non-steroidal anti-inflammatory drugs (NSAIDs) is their ability to stabilize or protect (prevent denaturation) heat-treated albumin at the physiological pH (pH: 6.2–6.5) [30].

The anti-inflammatory potential of seasonal SPE was evaluated in vitro by measuring its capacity to inhibit heat-induced BSA protein denaturation. The inhibitory effect of the extracts and diclofenac sodium (DS) at different concentrations (6.25–50 µg/mL) on protein denaturation is shown in Table 3. According to the results, seasonal SPE and DS were able to inhibit protein denaturation in a concentration-dependent manner. The percentage inhibition of protein denaturation of the seasonal SPE was within the range of 81.67% to 100% at the concentration range of 6.25 to 50 µg/mL, while DS presented its inhibitory effect within the range of 79.16% to 111.45% at the same concentration range. A previous study revealed that the hydroalcoholic extracts of Portuguese propolis were able to inhibit the denaturation of BSA protein by 24.93% to 74.69% [31], which are lower values than those obtained for the seasonal SPE (81.67% to 100%). Afonso et al. [32] also evaluated the anti-inflammatory potential of ethanolic extracts of Portuguese propolis and found that they inhibited protein denaturation by 28–45%. In general, all the extracts demonstrated similar behavior and, consequently, similar anti-inflammatory capability compared with DS, the commercial anti-inflammatory used as a drug reference. Among the SPE, the extract from W showed the highest inhibitory activity with a 100% inhibition of protein denaturation. Interestingly, at the lowest extract concentration tested (6.25 µg/mL), the extract from W and A exhibited a higher inhibitory effect than DS, indicating that these extracts could exert an anti-inflammatory effect at lower concentrations than those used for conventional anti-inflammatory drugs. The capacity of SPE flavonoids, chrysin, galangin, and pinocembrin to stabilize heat-treated BSA was also investigated (Table 3). In general, all the compounds showed less inhibitory activity of protein denaturation than seasonal SPE. Chrysin was the most effective compound to inhibit protein denaturation, with 70% inhibition at a concentration of 20 µM, followed by galangin and pinocembrin, with 60 and 57% of inhibition, respectively. This result coincides with the higher capacity of chrysin to decrease the NO levels in LPS-stimulated RAW 264.7 cells (Figure 2). According to the results, the capacity of the extracts to prevent protein denaturation could be partly attributed to a synergistic effect of the flavonoids and other components present in the extracts.

### 2.4. Effect of SPE and SPC on Hypotonicity-Induced Hemolysis of HRBC

**Figure 3:**
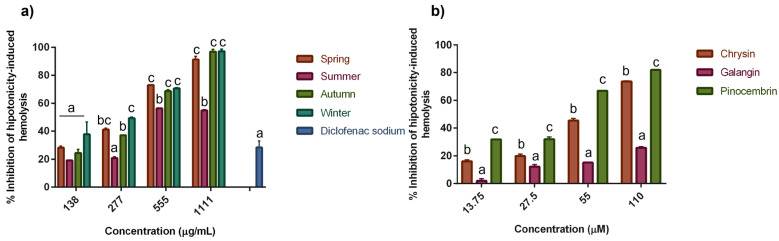
Effect of (**a**) seasonal SPE and (**b**) SPC on inhibition of hypotonicity-induced hemolysis. Values represent means ± SD of three independent experiments (*n* = 3). Bars with different letters within the same group of concentration indicate statistical differences (*p* ≤ 0.05).

Hypotonic solutions produce hemolysis, which refers to the accumulation of excessive fluids into cells resulting in the rupture of the red blood cells membrane. This injury increases the susceptibility of membrane cells to become damaged by free-radical-induced lipid peroxidation [33,34], with a subsequent increase of membrane permeability due to the participation of inflammatory intermediators [34]. The release of serum proteins and fluids into the tissue can be prevented by membrane stabilization. The percentage inhibition of hypotonicity-induced hemolysis of human red blood cells (HRBC) treated with seasonal SPE is shown in Figure 3a. In general, the extracts inhibited the hemolysis in a concentration-dependent manner. At the higher concentration evaluated, 1111 µg/mL, the percentage inhibition of each extract was 91, 54, 97, and 97% for S, M, A, and W, respectively, while DS, the drug used as a reference, showed a protection of 28% at the same concentration. According to the results, the extracts from S, A, and W exhibited similar (*p* ≤ 0.05) protection to the erythrocyte membrane at the two higher concentrations evaluated, 555 and 1111 µg/mL. Moreover, these treatments were the most effective in protecting the erythrocyte membrane against hypotonicity, with a percentage inhibition of hemolysis in the range of 68–73% and 91–97% at concentrations of 555 and 1111 µg/mL, respectively. The inhibition of hypotonicity-induced hemolysis (%) of SPC is shown in Figure 3b. All the compounds exhibited a protective effect against hemolysis in a dose-dependent manner. Pinocembrin showed the highest inhibitory effect, with 82% inhibition, followed by chrysin and galangin, with 74% and 26%, respectively. At all the concentrations tested, pinocembrin was the most effective compound to inhibit the hypotonicity-induced hemolysis, showing 32% inhibition at the lowest concentration tested (13.75 µM) and 82% inhibition at the highest concentration (110 µM). Chrysin also showed a considerable protective effect by inhibiting hemolysis by 16–74% at concentrations from 13.75 to 110 µM. The erythrocyte cell membrane is highly susceptible to lipid peroxidation by free-radical-mediated damage causing injury to the cell membrane integrity and leading to hemolysis. Some phytoflavonoids have been demonstrated to protect erythrocyte membranes from damage and inhibit hemolysis due to their antioxidant effect [35]. Oxidative stress usually causes membrane lipid peroxidation. Erythrocyte membranes are rich in unsaturated fats; moreover, hemoglobin in erythrocytes contains iron molecules. These characteristics make erythrocytes sensitive to oxidative damage from ROS [36]. Therefore, the elimination of excessive free radicals could reduce membrane lipid peroxidation and protect erythrocytes from oxidative injury. Flavonoids can scavenge free radicals, enhance reducing power, and inhibit lipid peroxidation through electron transfer or as hydrogen donors [35]. In addition, some flavonoids have metal-chelating potential, which allow them to scavenge the excess of metal ions in the body [37]. The antioxidant activity of flavonoids is closely related to their structural characteristics. Flavonoids usually contain one or more aromatic hydroxyl groups, which can react with free radicals to generate a stable semi-quinone structure, terminating the reaction of free radical chain [38]. AAPH (2,2 0-azobis (2-amidinopropane) dihydrochloride) is an alkyl radical generator that attacks erythrocyte membrane components and affects their structure and function, leading to erythrocyte hemolysis. A recent study demonstrated that flavonoids from mulberry leaves exert a protective effect against AAPH-induced hemolysis in sheep erythrocytes [35]. In addition, the authors observed a decrease in the malondialdehyde levels, which is an indicator of the degree of lipid peroxidation in AAPH-treated cells.

### 2.5. Effect of SPE and SPC on Heat-Induced Hemolysis of HRBC

The release of lysosomal contents of activated neutrophils, for instance, bacterial enzymes, and proteases, as a response to irritation, infection, or injury of living tissues, may damage macromolecules and cause lipid peroxidation of membranes, leading to tissue inflammation [39]. The HRBC membrane is analogous to the lysosomal membrane, and its stabilization by extracts or compounds implies that they may stabilize the lysosomal membranes. For this reason, HRBC membrane stabilization has been used as a method to examine the anti-inflammatory activity in vitro of extracts and compounds [39].

**Figure 4:**
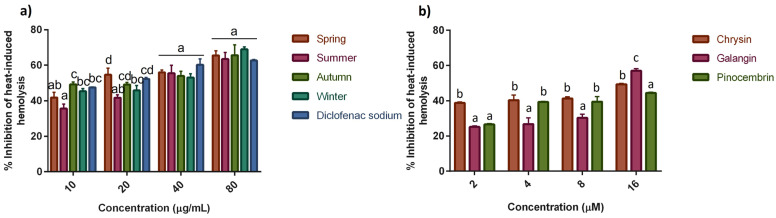
Effect of (**a**) seasonal SPE and (**b**) SPC on inhibition of heat-induced hemolysis. Values represent means ± SD of three independent experiments. Bars with different letters within the same group of concentration indicate statistical differences (*p* ≤ 0.05).

The percentage inhibition of heat-induced hemolysis of HRBC treated with different concentrations of seasonal SPE is shown in Figure 4. It was observed that all seasonal SPE were able to inhibit hemolysis in a concentration-dependent manner, showing an inhibition between 35% and 67%, with concentrations within the range of 10 to 80 µg/mL. At the maximum concentration analyzed, 80 µg/mL, the extracts showed 63–69% protection to heat-induced hemolysis, comparable to the standard drug, DS, with 62% inhibition of hemolysis. The latter provides evidence that the potential anti-inflammatory activity of seasonal SPE could be compared to some commonly used anti-inflammatory drugs. NSAIDs, such as DS, exert their action by inhibiting the enzyme cyclooxygenase. Unfortunately, these type of drugs have been associated with a high risk of acute myocardial infarction, stroke, heart failure, and arterial hypertension, among other issues [40]. The IC_50_ values of seasonal SPE were 21.68 ± 0.91, 40.77 ± 3.97, 18.94 ± 2.41, and 26.71 ± 0.54 µg/mL for S, M, A, and W, respectively. According to these results, the extracts from S, A, and W were found to be the most effective in protecting HRBC from heat-induced hemolysis, showing similar (*p* ˂ 0.05) IC_50_ values. In contrast, the less effective extract was M, as evidenced by its higher IC_50_ value. The capacity of seasonal SPE to protect HRBC membranes could be related to the presence of saponins and flavonoids, which have been reported to possess a good stabilizing effect on lysosomal membranes both in vivo and in vitro [41]. It has been suggested that the mode of action of extracts and anti-inflammatory drugs to stabilize membranes could be associated with their ability to bind the erythrocyte membranes with subsequent alteration of the surface charges of the cells. This modification on the membrane surface might prevent physical interaction with aggregating agents or promote dispersal by the mutual repulsion of similar charges involved in the hemolysis process of red blood cells [42]. Depending on their chemical structure, flavonoids may interact with membranes through two mechanisms: (1) the interaction between the hydrophobic nature of the flavonoid and the non-polar core of the membrane and (2) the interaction between hydrophilic flavonoids and the polar headgroups of lipids at the lipid–water interface, mainly through hydrogen bonds [43]. The interaction of flavonoids with the polar head of phospholipids can contribute to the rigidity of the membrane, reduce the fluidity, and increase the mechanical lipid bilayer stability [44]. Moreover, it has been suggested that the interaction of polyphenols at the bilayer’s surface by hydrogen bonding could reduce the access to harmful molecules, such as oxidants, thus protecting the structure and function of membranes [43]. In this sense, SPE could be contributing to stabilizing the erythrocyte membranes through the interaction of the flavonoids (SPC) present in the extracts with the lipid polar headgroups by protecting the bilayer against the aggression of deleterious molecules and increasing its mechanical stability. Regarding the ability of the compounds chrysin, galangin, and pinocembrin to inhibit heat-induced hemolysis, the maximum inhibition (%) observed was 57% for galangin, followed by 49 and 44% for chrysin and pinocembrin, respectively, at a concentration of 16 µM. The findings of the present study provide evidence for membrane stabilization as an additional mechanism of the anti-inflammatory potential of SPE, as the release of lysosomal contents from neutrophils might be inhibited at the inflammation site. Additionally, it was observed that under the conditions evaluated in this study, seasonality impacts the anti-inflammatory activity of SPE.

## 3. Materials and Methods

### 3.1. Propolis Samples Collection

Propolis was seasonally collected by the end of each season, from winter 2014 to fall 2015. Raw propolis samples were obtained from twelve hives of the “Tecolote” farm (N 31°02.18′, W 112°02.58′) located in the region of “El Arenoso”, between the municipalities of Caborca and Altar in the state of Sonora, Mexico. Previous studies have demonstrated the stability of the propolis chemical composition throughout the years [13]. Chrysin (purity by HPLC: 100%), galangin (purity by HPLC: 100%), and pinocembrin (purity by HPLC: 98%) were purchased from INDOFINE Chemical Co., Inc. (Hillsborough, NJ, USA).

### 3.2. Preparation and Characterization of Methanolic Extract of Propolis

Seasonal Sonoran propolis extracts (SPE) were prepared according to the previously reported method [13]. Small pieces of propolis samples (10 g) were mixed with methanol (50 mL) for 3–4 days with occasional stirring. Afterwards, the samples were filtered (Whatman filter paper No. 4) under reduced pressure to concentrate the extracts. Finally, the concentrated extracts were stored in the dark at −20 °C for further analysis. The polyphenolic profile of the major constituents of SPE was previously characterized [13,16] by high performance liquid chromatography (HPLC–UV–DAD) using an Agilent (Santa Clara, CA, USA) 1290 Infinite series equipped with ultraviolet and diode array detectors. The chemical standards of poplar compounds, pinocembrin, galangin, chrysin, and pinobanksin-3-O-acetate, were used to quantify poplar compounds in the samples. The calibration curves of each chemical standard were prepared from 0 to 80 µg/mL. The samples showed the following composition (Table 4):

### 3.3. Cell Culture Conditions

The RAW 264.7 cell line (TIB-71TM) was obtained from the American Type Culture Collection (ATCC; Rockville, MD, USA). Cells were cultured in Dulbecco Modified Eagle Medium (DMEM) supplemented with 5% fetal bovine serum (FBS), L-asparagine (98%), L-arginine monohydrochloride (≥98%), L-glutamine (200 mM), sodium pyruvate (100 mM), and penicillin–streptomycin solution (1000 U/1 U per mL) (D5F). Cells were maintained at 37 °C, 5% CO_2,_ and 98% relative humidity in an isothermal incubator (Thermo Fisher Scientific, Waltham, MA, USA).

### 3.4. Antiproliferative Activity Assay

The antiproliferative activity of SPE and the constituents (SPC) was determined using the MTT assay [45] with modifications [16]. In brief, RAW 264.7 cells (1 × 10^4^ cells, 50 µL) were seeded in a 96-well plate and incubated for 24 h at 37 °C and 5% CO_2_. Afterwards, aliquots of D5F containing different concentrations (1.5–50 µg/mL) of SPE or SPC (1.25–80 µM) were added to the wells and incubated for 48 h. Dimethyl sulfoxide (DMSO) was used as dissolvent control. In the last 4 h incubation, 10 µL of an MTT solution (5 mg/mL) was added to each well. At the end of incubation, the purple formazan crystals formed were dissolved with acidic isopropyl alcohol (0.4%, 100 µL). The absorbance was read at a test wavelength of 570 nm and a reference wavelength of 630 nm using an ELISA plate reader (Multiskan Go Thermo Scientific, Waltham, MA, USA). The results of antiproliferative activity were expressed as IC_50_ values obtained by linear regression analysis. The concentration of the extracts or compound where viability was ˂80% was considered cytotoxic.

### 3.5. Nitric Oxide Inhibitory Assay

The nitric oxide levels produced by RAW 264.7 cells exposed to SPE and SPC were determined by measuring nitrite in cell supernatants using the Griess reagent following the previously described method [46]. The cells (1 × 10^5^ cells/well, 100 µL) were seeded in a 96-well plate and incubated for 24 h at 37 °C and 5% CO_2_. After incubation, the cells were treated with 50 µL of SPE (10–1.25 µg/mL) or SPC (10–1.5 µM) in D5F and stimulated with 50 µL of lipopolysaccharide (LPS, 1 µg/mL) in D5F and further incubated for 24 h. Cells added with DMSO and with and without LPS were used as controls. At the end of incubation, aliquots (50 µL) of cell supernatants were collected and combined with an equal volume of Griess reagent (1% sulphanilamide/0.1% N-(1-napthyl) ethylene diamine (Sigma, St. Louis, MI, USA), each in 2.5% H_3_PO_4_) in a 96-well plate and incubated in the dark at room temperature for 10 min. Finally, the absorbance was read at 550 nm in an ELISA plate reader (Multiskan Go, Thermo Scientific, Waltham, MA, USA). A standard sodium nitrite curve was used to determine the NO levels. The results were reported as percentage of NO compared with the control. The IC_50_ values were determined by linear regression.

### 3.6. Inhibition of Heat-Induced Bovine Serum Albumin (BSA) Denaturation Assay

The protein denaturation assay was performed according to a modified method by Williams et al. [47]. In brief, stock solutions of different concentrations of SPE and SPC were prepared in methanol. Aliquots of the stocks (5 µL) were added to test tubes containing 500 µL of 0.4% (*w*/*v*) BSA buffer solution (pH 6.4, adjusted with glacial acetic acid). Diclofenac sodium and methanol were used as positive and negative controls, respectively. The reaction mixtures were heated at 70 °C for 10 min in a water bath and then cooled for 20 min at room temperature. The turbidity of solutions (level of protein denaturation) was measured at 660 nm using an ELISA plate reader (Multiskan Go, Thermo Scientific, Waltham, MA, USA). The percentage inhibition of precipitation (protein denaturation) was determined on a percentage basis relative to the negative control using the following equation:

% Anti-denaturation activity: [(Ac − As)/Ac] × 100

where Ac and As represent the absorbance of the control and the sample, respectively.

### 3.7. Preparation of Human Red Blood Cells (HRBC) Suspension

Fresh whole blood was obtained from a healthy human donor with prior informed consent. The sample was collected into EDTA tubes to prevent clotting. HRBC samples were resuspended in normal saline solution (0.9%, 1:4) and centrifuged at 2000× *g* for 10 min at 4 °C. The process was repeated three times until the supernatant was clear. The obtained packed cells were measured and reconstituted as a 10% *v*/*v* HRBC suspension with normal saline solution and used for the experiment [48].

### 3.8. Hypotonicity-Induced Hemolysis

The effect of SPE and SPC on HRBC stability was carried out as previously described [41,49] with few modifications. The assay mixture consisted of 200 µL of hypotonic (0.36% *w*/*v* NaCl) solution, 100 µL of PBS (10 mM, pH 7.4), 100 µL of SPE or SPC dissolved in normal saline solution, and 50 µL of 10% HRBC suspension. Two controls were prepared: one with normal saline solution instead of extract (Control 1) and one with 50 µL of normal saline solution instead of HRBC suspension (Control 2). Diclofenac sodium was used as a standard drug. The reaction mixtures were incubated at 56 °C for 30 min under stirring. After incubation, the tubes were centrifuged at 5000 rpm for 10 min at 4 °C. Finally, the supernatants were recovered, and the absorbance was read at 560 nm using an ELISA plate reader (Multiskan Go, Thermo Scientific, Waltham, MA, USA). The percentage of membrane stability was estimated using the equation explained by Sadique et al. [49], where the control represents 100% of HRBC lysis:

% Membrane stability = 100 − (As − Ac_2_/Ac_1_) × 100

where As denotes absorbance of sample; Ac_1_ and Ac_2_ denote absorbance of Control 1 and Control 2, respectively.

### 3.9. Heat-Induced Hemolysis

The HRBC suspension was reconstituted as a 10% (*v*/*v*) suspension in PBS (10 mM, pH 7.4). The assay was carried out as described by Shinde et al. [50] with some modifications by Gunathilake et al. [51]. The experiments were placed into two duplicate sets of centrifuge tubes. Briefly, 50 µL of HRBC suspension and 50 µL of hydromethanolic SPE or SPC at different concentrations were mixed with 2.95 mL of PBS. A pair of tubes containing PBS or diclofenac sodium instead of extract were used as controls. One pair of the tubes was incubated at 54 °C for 30 min under stirring, and the other pair was maintained at −10 °C for 30 min. The reaction mixtures were centrifuged at 2500 rpm for 3 min, and the absorbance of the supernatants was measured at 540 nm using an ELISA plate reader (Multiskan Go, Thermo Scientific, Waltham, MA, USA). The percentage inhibition of hemolysis was calculated according to the following equation [50]:

% Inhibition of hemolysis = 100 × [1− (A_2_ − A_1_/A_3_ − A_1_)]

where A_1_ denotes unheated test sample, A_2_ denotes heated test sample, and A_3_ denotes heated control sample.

### 3.10. Statistical Analysis

Values are expressed as mean ± SD of replicates. Data were analyzed by one-way ANOVA, and mean difference between means were determined by the Tukey–Kramer test (*p* ≤ 0.05) using Prism 7 for Windows software (GraphPad Software, 2016, San Diego, CA, USA).

## 4. Conclusions

The results indicate that the seasonal Sonoran propolis extracts possess cytotoxic and anti-inflammatory activities, depending on dose and collection time. Moreover, the findings indicate that the anti-inflammatory potential of Sonoran propolis is directly influenced by the variations in its chemical composition and that the flavonoids chrysin, galangin, and pinocembrin could be partly contributing to this property through a synergistic effect, likely involving other unidentified compounds. The anti-inflammatory assays showed that seasonal Sonoran propolis extracts were able to reduce the NO levels to basal levels on RAW 264.7 cells, inhibit the heat-induced protein denaturation, and protect the HRBC membrane from hemolysis induced by heat and hypotonicity, which could be considered as possible mechanisms of Sonoran propolis to exert its anti-inflammatory effect. The results obtained in the present study reveal that Sonoran propolis possesses a potent pharmacological potential and that some of their constituents could be promising candidates for developing new anti-inflammatory drugs.

## Figures and Tables

**Table 1:** Antiproliferative activity of seasonal Sonoran Propolis Extracts and Sonoran Popolis Constituents on RAW 264.7 cells.

Sample	IC_50_ (µg/mL or µM)
Spring (S)	26.60 ± 2.00 ^b^
Summer (M)	49.40 ± 1.80 ^a^
Autumn (A)	26.50 ± 1.50 ^b^
Winter (W)	30.20 ± 2.00 ^b^
Chrysin	56.20 ± 1.10 ^a^
Galangin	52.40 ± 0.50 ^a^
Pinocembrin	56.16 ± 2.30 ^a^

IC_50_ values of SPE (µg/mL) and SPC (µM) represent the mean ± SD of at least three independent experiments performed in triplicate (*n* = 3). Mean values with different superscripts indicate statistical differences (*p* ≤ 0.05). IC_50_ value corresponds to the concentration that inhibits 50% of cell proliferation.

**Table 2:** Inhibition of the NO production in RAW 264.7 cells treated with seasonal Sonoran Propolis Extracts and Sonoran Propolis Constituents.

Sample	IC_50_ (µg/mL or µM)
Spring (S)	3.35 ± 0.30 ^a^
Summer (M)	8.68 ± 0.01 ^d^
Autumn (A)	4.59 ± 0.02 ^b^
Winter (W)	5.80 ± 0.04 ^c^
Chrysin	5.80 ± 0.20
Galangin	>10
Pinocembrin	>10

IC_50_ values of SPE (µg/mL) and SPC (µM) represent the mean ± SD of at least three independent experiments performed in triplicate (*n* = 3). Mean values with different superscripts indicate statistical differences (*p* ≤ 0.05). IC_50_ value corresponds to the concentration that inhibits 50% of the NO production.

**Table 3:** Effect of seasonal Sonoran Propolis Extracts and Sonoran Propolis Constituents on heat-induced BSA denaturation.

**Concentration** **(** **µ** **g/mL)**	**Spring**	**Summer**	**Autumn**	**Winter**	**Diclofenac Sodium**
	Inhibition (%)				
50	95.56 ± 1.62 ^a^	93.40 ± 3.75 ^a^	100.00 ± 2.94 ^b^	93.89 ± 3.49 ^a^	111.45 ± 1.47 ^c^
25	86.26 ± 0.00 ^b^	91.03 ± 1.88 ^c^	93.12 ± 3.96 ^d^	89.69 ± 1.61 ^c^	83.33 ± 2.94 ^a^
12.5	86.20 ± 0.00 ^a^	90.35 ± 1.61 ^b^	91.04 ± 2.20 ^b^	87.02 ± 1.32 ^a^	85.41 ± 5.89 ^a^
6.25	81.67 ± 3.23 ^a^	83.02 ± 1.90 ^a^	93.89 ± 1.22 ^c^	89.31 ± 1.32 ^b^	79.16 ± 0.98 ^a^
**Concentration** **(** **µM)**	**Chrysin**		**Galangin**		**Pinocembrin**
	Inhibition (%)				
20	76.20 ± 1.18 ^c^		60.33 ± 0.16 ^b^		57.36 ± 1.43 ^a^
10	38.04 ± 1.58 ^c^		25.84 ± 1.69 ^a^		35.02 ± 1.43 ^b^
5	28.36 ± 1.52 ^b^		12.5 ± 1.35 ^a^		31.97 ± 1.43 ^c^
2.5	26.34 ± 1.49 ^c^		11.16 ± 1.13 ^a^		15.14 ± 0.74 ^b^

Data are expressed as means ± SD of three independent experiments (*n* = 3). Mean values with different superscripts in the same line indicate statistical differences (*p* ≤ 0.05).

**Table 4:** Polyphenolic composition of seasonal Sonoran propolis extracts.

Compound	Concentration (µg/mg)			
	Spring	Summer	Autumn	Winter
Pinocembrin	12.80 ± 2.30	45.80 ± 3.80	19.10 ± 3.00	30.10 ± 0.20
Pinobanksin-3-O-acetate	˂5.00	24.90 ± 0.50	9.10 ± 0.10	13.70 ± 1.50
Chrysin	˂5.00	˂5.00	˂5.00	˂5.00
Galangin	˂5.00	˂5.00	˂5.00	7.40 ± 0.60

Adapted from: Mendez-Pfeiffer et al. [13].

## Data Availability

The original contributions data presented in this research are included in the article; further inquiries can be directed to the corresponding author. The data are not publicly available due to the fact that the author of correspondence keeps control of its diffusion by regulations of the University of Sonora; however, the information can be requested without problem from the people who require it.
